# A nonmerohedral twin of methyl 2-[meth­yl(methyl­sulfon­yl)amino]benzoate

**DOI:** 10.1107/S1600536809037799

**Published:** 2009-09-30

**Authors:** Muhammad Shafiq, Islam Ullah Khan, Reza Kia, Muhammad Nadeem Arshad, Maooz Aslam

**Affiliations:** a Materials Chemistry Laboratory, Department of Chemistry, GC University, Lahore 54000, Pakistan; b Department of Chemistry, Science and Research Campus, Islamic Azad University, Poonak, Tehran, Iran

## Abstract

The asymmetric unit of the title compound, C_10_H_13_NO_4_S, comprises two crystallographically independent mol­ecules. The crystal structure is stabilized by weak inter­molecular C—H⋯O hydrogen bonds, which link mol­ecules along the *b* axis. The crystal is a nonmerohedral twin, the refined ratio of the twin components being 0.344 (2):0.656 (2).

## Related literature

For standard values of bond lengths, see: Allen *et al.* (1987 ▶). For applications of benzothia­zine derivatives in organic synthesis, see: Shafiq *et al.* (2008 ▶, 2009*a* ▶,*b* ▶); Lombardino (1972 ▶); Arshad *et al.* (2008 ▶).
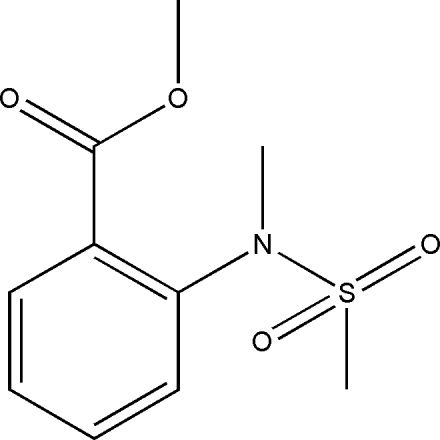


## Experimental

### 

#### Crystal data

C_10_H_13_NO_4_S
*M* *_r_* = 243.27
Monoclinic, 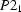

*a* = 8.7476 (4) Å
*b* = 10.2081 (4) Å
*c* = 13.8377 (7) Å
β = 108.347 (3)°
*V* = 1172.84 (9) Å^3^
*Z* = 4
Mo *K*α radiation
μ = 0.27 mm^−1^
*T* = 296 K
0.32 × 0.21 × 0.15 mm

#### Data collection

Bruker SMART APEXII CCD area-detector diffractometer
Absorption correction: multi-scan (**SADABS**; Bruker, 2005 ▶) *T*_min_ = 0.917, *T*_max_ = 0.960
13537 measured reflections
5720 independent reflections
3842 reflections with *I* > 2σ(*I*)
*R*_int_ = 0.041

#### Refinement

*R*[*F* ^2^ > 2σ(*F* ^2^)] = 0.043
*wR*(*F* ^2^) = 0.083
*S* = 0.99
5720 reflections
296 parameters
1 restraint
H-atom parameters constrained
Δρ_max_ = 0.20 e Å^−3^
Δρ_min_ = −0.29 e Å^−3^
Absolute structure: Flack (1983), 2643 Friedel pairs
Flack parameter: 0.00 (8)

### 

Data collection: *APEX2* (Bruker, 2005 ▶); cell refinement: *SAINT* (Bruker, 2005 ▶); data reduction: *SAINT*; program(s) used to solve structure: *SHELXTL* (Sheldrick, 2008 ▶); program(s) used to refine structure: *SHELXTL*; molecular graphics: *SHELXTL*; software used to prepare material for publication: *SHELXTL* and *PLATON* (Spek, 2009 ▶).

## Supplementary Material

Crystal structure: contains datablocks global, I. DOI: 10.1107/S1600536809037799/lh2905sup1.cif

Structure factors: contains datablocks I. DOI: 10.1107/S1600536809037799/lh2905Isup2.hkl

Additional supplementary materials:  crystallographic information; 3D view; checkCIF report

## Figures and Tables

**Table 1:** Hydrogen-bond geometry (Å, °)

*D*—H⋯*A*	*D*—H	H⋯*A*	*D*⋯*A*	*D*—H⋯*A*
C13—H13⋯O14^i^	0.93	2.58	3.248 (6)	129
C20—H20*C*⋯O11^ii^	0.96	2.29	3.240 (5)	171
C22—H22⋯O24^iii^	0.93	2.51	3.391 (5)	159
C28—H28*A*⋯O13^iv^	0.96	2.54	3.490 (6)	169
C30—H30*A*⋯O14^v^	0.96	2.55	3.512 (5)	178
C30—H30*B*⋯O21^iii^	0.96	2.42	3.181 (6)	136

Symmetry codes: (i) 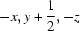; (ii) 
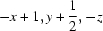
; (iii) 
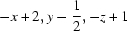
; (iv) 
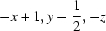
; (v) 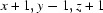.

**Fig. 1.**
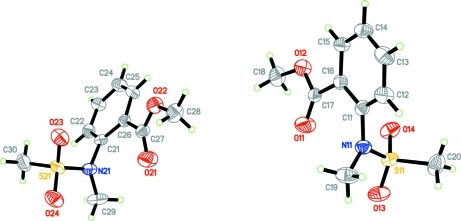
The molecular structure of the title compound, showing 30% probability displacement ellipsoids and the atomic numbering.

**Fig. 2.**
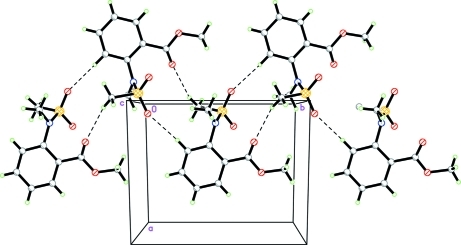
The crystal packing of the title compound, viewed down the c-axis, showing linking of the molecules through intermolecular C—H···O interactions along the b-axis. Intermolecular interactions are drawn as dashed lines.

## supplementary crystallographic information

### Comment

Our group is actively involved in the synthesis and characterization by X-ray studies of various benzothiazine derivatives (Shafiq *et al.*, 2009*a*; Shafiq *et al.*,  2009*b*; Shafiq *et al.*,  2008; Arshad *et al.*,  2008). The crystal structure of the title compound is reported here.

In the molecule of the title compound, (Fig. 1), intermolecular C—H···O interactions (Table 1) link neighbouring molecules along the *b* axis (Fig. 2). The crystal was a nonmerohedral twin with a refined BASF parameter ratio of 0.344 (2)/0.656 (2).

### Experimental

The title compound was synthesized as reported earlier (Lombardino *et al.*, 1972) and was recrystallized from a solution of Ethanol by slow evaporation at room temperature.

### Refinement

All of the hydrogen atoms were positioned geometrically [C—H = 0.93–0.96 Å] and refined using a riding model approximation with U_iso_ (H) = 1.2 or 1.5 U_eq_ (C). A rotating group model was applied for the methyl groups. PLATON and intensity statistic indicate nonmerohedral twinning. Applying the twin instruction [TWIN -1 0 0 0 -1 0 1 0 1] with a BASF parameter in SHELXLTL (Sheldrick, 2008) of 0.365 (1) in SHELXTL (Sheldrick, 2008), the R1 value drops to 0.043 (0.1886 without TWIN instruction).

### Crystal data

**Table:**

C_10_H_13_NO_4_S	*F*(000) = 512
*M**_r_* = 243.27	*D*_x_ = 1.378 Mg m^−^^3^
Monoclinic, *P*2_1_	Mo *K*α radiation, λ = 0.71073 Å
Hall symbol: P 2yb	Cell parameters from 3745 reflections
*a* = 8.7476 (4) Å	θ = 2.5–29.5°
*b* = 10.2081 (4) Å	µ = 0.27 mm^−^^1^
*c* = 13.8377 (7) Å	*T* = 296 K
β = 108.347 (3)°	Block, colourless
*V* = 1172.84 (9) Å^3^	0.32 × 0.21 × 0.15 mm
*Z* = 4	

### Data collection

**Table:**

Bruker SMART APEXII CCD area-detector diffractometer	5720 independent reflections
Radiation source: fine-focus sealed tube	3842 reflections with *I* > 2σ(*I*)
graphite	*R*_int_ = 0.041
φ and ω scans	θ_max_ = 28.3°, θ_min_ = 1.6°
Absorption correction: multi-scan (*SADABS*; Bruker, 2005)	*h* = −11→11
*T*_min_ = 0.917, *T*_max_ = 0.960	*k* = −13→13
13537 measured reflections	*l* = −18→18

### Refinement

**Table:**

Refinement on *F*^2^	Secondary atom site location: difference Fourier map
Least-squares matrix: full	Hydrogen site location: inferred from neighbouring sites
*R*[*F*^2^ > 2σ(*F*^2^)] = 0.043	H-atom parameters constrained
*wR*(*F*^2^) = 0.083	*w* = 1/[σ^2^(*F*_o_^2^) + (0.0315*P*)^2^] where *P* = (*F*_o_^2^ + 2*F*_c_^2^)/3
*S* = 0.99	(Δ/σ)_max_ < 0.001
5720 reflections	Δρ_max_ = 0.20 e Å^−^^3^
296 parameters	Δρ_min_ = −0.29 e Å^−^^3^
1 restraint	Absolute structure: Flack (1983), 2643 Friedel pairs
Primary atom site location: structure-invariant direct methods	Flack parameter: 0.00 (8)

### Special details

**Table:**

Geometry. All esds (except the esd in the dihedral angle between two l.s. planes) are estimated using the full covariance matrix. The cell esds are taken into account individually in the estimation of esds in distances, angles and torsion angles; correlations between esds in cell parameters are only used when they are defined by crystal symmetry. An approximate (isotropic) treatment of cell esds is used for estimating esds involving l.s. planes.
Refinement. Refinement of F^2^ against ALL reflections. The weighted R-factor wR and goodness of fit S are based on F^2^, conventional R-factors R are based on F, with F set to zero for negative F^2^. The threshold expression of F^2^ > 2sigma(F^2^) is used only for calculating R-factors(gt) etc. and is not relevant to the choice of reflections for refinement. R-factors based on F^2^ are statistically about twice as large as those based on F, and R- factors based on ALL data will be even larger.

### Fractional atomic coordinates and isotropic or equivalent isotropic displacement parameters (Å^2^)

**Table:**

	*x*	*y*	*z*	*U*_iso_*/*U*_eq_	
S11	0.34203 (10)	1.01678 (10)	−0.09098 (6)	0.0492 (2)	
O13	0.4796 (4)	0.9599 (3)	−0.1065 (2)	0.0757 (9)	
O11	0.3556 (4)	0.8101 (3)	0.1014 (3)	0.0921 (12)	
O12	0.1243 (4)	0.7697 (2)	0.1261 (2)	0.0569 (8)	
O14	0.1950 (3)	0.9443 (3)	−0.1159 (2)	0.0631 (8)	
N11	0.3887 (3)	1.0581 (3)	0.0270 (2)	0.0460 (7)	
C11	0.2621 (5)	1.0872 (4)	0.0698 (3)	0.0440 (10)	
C12	0.2224 (5)	1.2160 (4)	0.0785 (3)	0.0569 (12)	
H12	0.2787	1.2817	0.0575	0.068*	
C13	0.1001 (6)	1.2502 (4)	0.1178 (4)	0.0679 (14)	
H13	0.0772	1.3377	0.1256	0.081*	
C14	0.0131 (5)	1.1521 (4)	0.1450 (3)	0.0551 (11)	
H14	−0.0731	1.1733	0.1678	0.066*	
C15	0.0540 (4)	1.0221 (5)	0.1385 (3)	0.0503 (8)	
H15	−0.0034	0.9566	0.1586	0.060*	
C16	0.1787 (4)	0.9889 (3)	0.1025 (3)	0.0431 (9)	
C17	0.2342 (5)	0.8487 (4)	0.1083 (3)	0.0491 (10)	
C18	0.1680 (6)	0.6327 (4)	0.1398 (3)	0.0737 (14)	
H18A	0.2723	0.6243	0.1898	0.111*	
H18B	0.1708	0.5966	0.0763	0.111*	
H18C	0.0899	0.5864	0.1623	0.111*	
C19	0.5524 (5)	1.0955 (5)	0.0835 (3)	0.0745 (14)	
H19A	0.5862	1.0495	0.1472	0.112*	
H19B	0.5567	1.1882	0.0960	0.112*	
H19C	0.6225	1.0737	0.0447	0.112*	
C20	0.3003 (6)	1.1605 (4)	−0.1635 (3)	0.0791 (15)	
H20A	0.2236	1.2121	−0.1435	0.119*	
H20B	0.2567	1.1385	−0.2343	0.119*	
H20C	0.3978	1.2098	−0.1524	0.119*	
S21	0.93347 (9)	0.01388 (10)	0.56230 (7)	0.0483 (2)	
O21	0.7083 (4)	0.2046 (3)	0.3383 (3)	0.0857 (10)	
O22	0.4802 (4)	0.2520 (3)	0.3686 (2)	0.0646 (9)	
O23	0.8215 (4)	0.0862 (3)	0.5968 (2)	0.0686 (9)	
O24	1.0850 (3)	0.0697 (3)	0.5679 (3)	0.0782 (10)	
N21	0.8462 (3)	−0.0237 (2)	0.4449 (2)	0.0460 (7)	
C21	0.6795 (4)	−0.0606 (4)	0.4145 (3)	0.0395 (9)	
C22	0.6397 (5)	−0.1920 (4)	0.4206 (3)	0.0493 (10)	
H22	0.7207	−0.2544	0.4423	0.059*	
C23	0.4821 (5)	−0.2289 (4)	0.3946 (3)	0.0522 (11)	
H23	0.4563	−0.3171	0.3963	0.063*	
C24	0.3619 (5)	−0.1381 (4)	0.3663 (3)	0.0591 (11)	
H24	0.2551	−0.1640	0.3515	0.071*	
C25	0.3989 (4)	−0.0073 (4)	0.3595 (3)	0.0520 (10)	
H25	0.3166	0.0544	0.3403	0.062*	
C26	0.5591 (4)	0.0328 (4)	0.3811 (2)	0.0402 (8)	
C27	0.5960 (5)	0.1720 (4)	0.3634 (3)	0.0488 (10)	
C28	0.5045 (7)	0.3895 (4)	0.3499 (4)	0.0806 (15)	
H28A	0.5186	0.3989	0.2842	0.121*	
H28B	0.5987	0.4213	0.4014	0.121*	
H28C	0.4123	0.4392	0.3518	0.121*	
C29	0.9422 (6)	−0.0656 (6)	0.3808 (4)	0.0864 (17)	
H29A	0.8828	−0.0498	0.3106	0.130*	
H29B	0.9657	−0.1574	0.3909	0.130*	
H29C	1.0411	−0.0169	0.3989	0.130*	
C30	0.9703 (5)	−0.1321 (5)	0.6318 (3)	0.0742 (14)	
H30A	1.0313	−0.1138	0.7013	0.111*	
H30B	1.0302	−0.1907	0.6030	0.111*	
H30C	0.8697	−0.1720	0.6293	0.111*	

### Atomic displacement parameters (Å^2^)

**Table:**

	*U*^11^	*U*^22^	*U*^33^	*U*^12^	*U*^13^	*U*^23^
S11	0.0494 (5)	0.0530 (6)	0.0502 (5)	−0.0082 (6)	0.0229 (4)	−0.0054 (6)
O13	0.079 (2)	0.082 (2)	0.084 (2)	0.0002 (16)	0.0516 (19)	−0.0212 (17)
O11	0.092 (3)	0.0563 (18)	0.157 (4)	0.0362 (18)	0.081 (3)	0.038 (2)
O12	0.0624 (19)	0.0402 (17)	0.070 (2)	−0.0011 (14)	0.0241 (16)	−0.0016 (13)
O14	0.0620 (18)	0.0702 (18)	0.0598 (17)	−0.0278 (15)	0.0230 (16)	−0.0191 (14)
N11	0.0370 (16)	0.0516 (18)	0.0512 (17)	−0.0085 (14)	0.0165 (15)	−0.0038 (12)
C11	0.045 (2)	0.045 (2)	0.041 (2)	−0.0018 (19)	0.0122 (18)	−0.0017 (15)
C12	0.058 (3)	0.041 (2)	0.073 (3)	−0.001 (2)	0.023 (2)	−0.007 (2)
C13	0.066 (3)	0.056 (3)	0.082 (4)	0.012 (3)	0.023 (3)	−0.017 (2)
C14	0.050 (2)	0.062 (3)	0.058 (2)	0.008 (2)	0.024 (2)	−0.010 (2)
C15	0.0497 (19)	0.053 (2)	0.054 (2)	−0.003 (3)	0.0241 (16)	−0.002 (2)
C16	0.047 (2)	0.042 (2)	0.0415 (18)	0.0067 (17)	0.0166 (17)	0.0016 (16)
C17	0.055 (3)	0.050 (2)	0.045 (2)	0.013 (2)	0.021 (2)	0.0116 (18)
C18	0.091 (4)	0.042 (2)	0.087 (3)	−0.002 (2)	0.025 (3)	0.010 (2)
C19	0.043 (2)	0.106 (4)	0.074 (3)	−0.017 (2)	0.018 (2)	−0.035 (3)
C20	0.078 (4)	0.077 (3)	0.075 (3)	−0.022 (3)	0.013 (3)	0.024 (3)
S21	0.0360 (5)	0.0431 (5)	0.0628 (5)	−0.0009 (5)	0.0111 (4)	−0.0045 (6)
O21	0.071 (2)	0.0638 (18)	0.134 (3)	−0.0058 (17)	0.048 (2)	0.026 (2)
O22	0.077 (2)	0.0438 (17)	0.080 (2)	0.0108 (16)	0.0350 (19)	0.0115 (14)
O23	0.0614 (19)	0.0662 (18)	0.075 (2)	0.0129 (16)	0.0176 (16)	−0.0248 (14)
O24	0.0447 (17)	0.071 (2)	0.107 (2)	−0.0199 (14)	0.0075 (17)	−0.0016 (16)
N21	0.0432 (17)	0.0425 (17)	0.0579 (17)	0.0006 (13)	0.0240 (16)	0.0025 (12)
C21	0.039 (2)	0.041 (2)	0.044 (2)	−0.0028 (17)	0.0205 (18)	−0.0020 (15)
C22	0.059 (3)	0.038 (2)	0.048 (2)	0.006 (2)	0.014 (2)	−0.0005 (16)
C23	0.054 (3)	0.038 (2)	0.065 (3)	−0.006 (2)	0.019 (2)	−0.0047 (19)
C24	0.046 (2)	0.058 (3)	0.073 (3)	−0.017 (2)	0.017 (2)	−0.009 (2)
C25	0.0394 (19)	0.055 (3)	0.059 (2)	0.004 (2)	0.0128 (17)	0.003 (2)
C26	0.0406 (19)	0.042 (2)	0.0377 (16)	−0.0059 (19)	0.0124 (15)	0.0013 (16)
C27	0.051 (3)	0.047 (2)	0.050 (2)	−0.001 (2)	0.020 (2)	0.0028 (18)
C28	0.115 (4)	0.041 (2)	0.098 (3)	0.012 (3)	0.050 (3)	0.015 (2)
C29	0.068 (3)	0.109 (4)	0.107 (4)	−0.010 (3)	0.061 (3)	−0.026 (3)
C30	0.066 (3)	0.080 (3)	0.064 (3)	0.007 (3)	0.002 (2)	0.014 (3)

### Geometric parameters (Å, °)

**Table:**

S11—O13	1.412 (3)	S21—O24	1.422 (3)
S11—O14	1.429 (3)	S21—O23	1.424 (3)
S11—N11	1.609 (3)	S21—N21	1.609 (3)
S11—C20	1.750 (4)	S21—C30	1.748 (5)
O11—C17	1.166 (5)	O21—C27	1.189 (4)
O12—C17	1.335 (5)	O22—C27	1.320 (4)
O12—C18	1.446 (5)	O22—C28	1.455 (5)
N11—C11	1.441 (5)	N21—C21	1.435 (4)
N11—C19	1.450 (5)	N21—C29	1.464 (5)
C11—C12	1.375 (5)	C21—C26	1.388 (5)
C11—C16	1.398 (5)	C21—C22	1.394 (5)
C12—C13	1.389 (6)	C22—C23	1.364 (6)
C12—H12	0.9300	C22—H22	0.9300
C13—C14	1.380 (6)	C23—C24	1.363 (6)
C13—H13	0.9300	C23—H23	0.9300
C14—C15	1.385 (6)	C24—C25	1.383 (6)
C14—H14	0.9300	C24—H24	0.9300
C15—C16	1.377 (5)	C25—C26	1.399 (5)
C15—H15	0.9300	C25—H25	0.9300
C16—C17	1.505 (5)	C26—C27	1.495 (5)
C18—H18A	0.9600	C28—H28A	0.9600
C18—H18B	0.9600	C28—H28B	0.9600
C18—H18C	0.9600	C28—H28C	0.9600
C19—H19A	0.9600	C29—H29A	0.9600
C19—H19B	0.9600	C29—H29B	0.9600
C19—H19C	0.9600	C29—H29C	0.9600
C20—H20A	0.9600	C30—H30A	0.9600
C20—H20B	0.9600	C30—H30B	0.9600
C20—H20C	0.9600	C30—H30C	0.9600
			
O13—S11—O14	119.71 (18)	O24—S21—O23	120.1 (2)
O13—S11—N11	107.81 (17)	O24—S21—N21	106.75 (18)
O14—S11—N11	107.60 (15)	O23—S21—N21	107.73 (16)
O13—S11—C20	106.9 (2)	O24—S21—C30	107.7 (2)
O14—S11—C20	106.7 (2)	O23—S21—C30	106.6 (2)
N11—S11—C20	107.6 (2)	N21—S21—C30	107.4 (2)
C17—O12—C18	115.6 (3)	C27—O22—C28	115.4 (3)
C11—N11—C19	118.7 (3)	C21—N21—C29	118.1 (3)
C11—N11—S11	119.2 (2)	C21—N21—S21	118.3 (2)
C19—N11—S11	120.5 (3)	C29—N21—S21	120.1 (3)
C12—C11—C16	119.1 (4)	C26—C21—C22	120.2 (3)
C12—C11—N11	118.8 (4)	C26—C21—N21	120.8 (3)
C16—C11—N11	122.1 (3)	C22—C21—N21	119.0 (3)
C11—C12—C13	121.4 (4)	C23—C22—C21	120.0 (4)
C11—C12—H12	119.3	C23—C22—H22	120.0
C13—C12—H12	119.3	C21—C22—H22	120.0
C14—C13—C12	119.0 (4)	C24—C23—C22	120.8 (4)
C14—C13—H13	120.5	C24—C23—H23	119.6
C12—C13—H13	120.5	C22—C23—H23	119.6
C13—C14—C15	120.1 (4)	C23—C24—C25	120.0 (4)
C13—C14—H14	120.0	C23—C24—H24	120.0
C15—C14—H14	120.0	C25—C24—H24	120.0
C16—C15—C14	120.7 (4)	C24—C25—C26	120.5 (4)
C16—C15—H15	119.7	C24—C25—H25	119.7
C14—C15—H15	119.7	C26—C25—H25	119.7
C15—C16—C11	119.6 (3)	C21—C26—C25	118.3 (4)
C15—C16—C17	119.6 (4)	C21—C26—C27	121.8 (3)
C11—C16—C17	120.5 (3)	C25—C26—C27	119.8 (4)
O11—C17—O12	122.6 (4)	O21—C27—O22	124.2 (4)
O11—C17—C16	127.2 (4)	O21—C27—C26	123.7 (4)
O12—C17—C16	110.2 (3)	O22—C27—C26	111.7 (3)
O12—C18—H18A	109.5	O22—C28—H28A	109.5
O12—C18—H18B	109.5	O22—C28—H28B	109.5
H18A—C18—H18B	109.5	H28A—C28—H28B	109.5
O12—C18—H18C	109.5	O22—C28—H28C	109.5
H18A—C18—H18C	109.5	H28A—C28—H28C	109.5
H18B—C18—H18C	109.5	H28B—C28—H28C	109.5
N11—C19—H19A	109.5	N21—C29—H29A	109.5
N11—C19—H19B	109.5	N21—C29—H29B	109.5
H19A—C19—H19B	109.5	H29A—C29—H29B	109.5
N11—C19—H19C	109.5	N21—C29—H29C	109.5
H19A—C19—H19C	109.5	H29A—C29—H29C	109.5
H19B—C19—H19C	109.5	H29B—C29—H29C	109.5
S11—C20—H20A	109.5	S21—C30—H30A	109.5
S11—C20—H20B	109.5	S21—C30—H30B	109.5
H20A—C20—H20B	109.5	H30A—C30—H30B	109.5
S11—C20—H20C	109.5	S21—C30—H30C	109.5
H20A—C20—H20C	109.5	H30A—C30—H30C	109.5
H20B—C20—H20C	109.5	H30B—C30—H30C	109.5
			
O13—S11—N11—C11	−164.2 (3)	O24—S21—N21—C21	−169.1 (3)
O14—S11—N11—C11	−33.8 (3)	O23—S21—N21—C21	−38.9 (3)
C20—S11—N11—C11	80.8 (3)	C30—S21—N21—C21	75.5 (3)
O13—S11—N11—C19	30.1 (4)	O24—S21—N21—C29	32.8 (3)
O14—S11—N11—C19	160.5 (3)	O23—S21—N21—C29	163.0 (3)
C20—S11—N11—C19	−84.9 (4)	C30—S21—N21—C29	−82.6 (3)
C19—N11—C11—C12	67.4 (5)	C29—N21—C21—C26	−111.5 (4)
S11—N11—C11—C12	−98.6 (4)	S21—N21—C21—C26	89.9 (3)
C19—N11—C11—C16	−112.7 (4)	C29—N21—C21—C22	69.5 (5)
S11—N11—C11—C16	81.3 (4)	S21—N21—C21—C22	−89.1 (3)
C16—C11—C12—C13	−0.8 (6)	C26—C21—C22—C23	−0.7 (6)
N11—C11—C12—C13	179.1 (4)	N21—C21—C22—C23	178.3 (3)
C11—C12—C13—C14	−2.5 (7)	C21—C22—C23—C24	−2.5 (7)
C12—C13—C14—C15	3.7 (7)	C22—C23—C24—C25	2.7 (7)
C13—C14—C15—C16	−1.7 (6)	C23—C24—C25—C26	0.2 (6)
C14—C15—C16—C11	−1.6 (5)	C22—C21—C26—C25	3.5 (5)
C14—C15—C16—C17	172.4 (3)	N21—C21—C26—C25	−175.5 (3)
C12—C11—C16—C15	2.8 (5)	C22—C21—C26—C27	−173.6 (3)
N11—C11—C16—C15	−177.1 (3)	N21—C21—C26—C27	7.4 (5)
C12—C11—C16—C17	−171.1 (4)	C24—C25—C26—C21	−3.3 (5)
N11—C11—C16—C17	9.0 (5)	C24—C25—C26—C27	173.9 (4)
C18—O12—C17—O11	2.1 (6)	C28—O22—C27—O21	−5.3 (6)
C18—O12—C17—C16	−176.3 (3)	C28—O22—C27—C26	−178.7 (4)
C15—C16—C17—O11	−162.8 (4)	C21—C26—C27—O21	29.6 (6)
C11—C16—C17—O11	11.2 (7)	C25—C26—C27—O21	−147.4 (4)
C15—C16—C17—O12	15.5 (5)	C21—C26—C27—O22	−157.0 (3)
C11—C16—C17—O12	−170.6 (3)	C25—C26—C27—O22	26.0 (5)

### Hydrogen-bond geometry (Å, °)

**Table:**

*D*—H···*A*	*D*—H	H···*A*	*D*···*A*	*D*—H···*A*
C13—H13···O14^i^	0.93	2.58	3.248 (6)	129
C20—H20C···O11^ii^	0.96	2.29	3.240 (5)	171
C22—H22···O24^iii^	0.93	2.51	3.391 (5)	159
C28—H28A···O13^iv^	0.96	2.54	3.490 (6)	169
C30—H30A···O14^v^	0.96	2.55	3.512 (5)	178
C30—H30B···O21^iii^	0.96	2.42	3.181 (6)	136

Symmetry codes: (i) −*x*, *y*+1/2, −*z*; (ii) −*x*+1, *y*+1/2, −*z*; (iii) −*x*+2, *y*−1/2, −*z*+1; (iv) −*x*+1, *y*−1/2, −*z*; (v) *x*+1, *y*−1, *z*+1.
